# Neutrophil Subsets in Periodontal Health and Disease: A Mini Review

**DOI:** 10.3389/fimmu.2019.03001

**Published:** 2020-01-08

**Authors:** Josefine Hirschfeld

**Affiliations:** Department of Periodontology, Birmingham Dental School and Hospital, Birmingham, United Kingdom

**Keywords:** phenotypal marker, cell function and behavior, subset, neutrophils, cluster of differentiation, periodontitis

## Abstract

Neutrophils are amongst the most abundant immune cells within the periodontal tissues and oral cavity. As innate immune cells, they are first line defenders at the tooth-mucosa interface, and can perform an array of different functions. With regard to these, it has been observed over many years that neutrophils are highly heterogeneous in their behavior. Therefore, it has been speculated that neutrophils, similarly to other leukocytes, exist in distinct subsets. Several studies have investigated different markers of neutrophils in oral health and disease in recent years in order to define potential cell subsets and their specific tasks. This research was inspired by recent advancements in other fields of medicine in this field. The aim of this review is to give an overview of the current evidence regarding the existence and presence of neutrophil subsets and their possible functions, specifically in the context of periodontitis, gingivitis, and periodontal health.

## Introduction

Neutrophils are found in high abundance within the periodontal tissues and oral cavity. They typically migrate from local capillaries within the periodontal tissues toward the gingival crevice, following the highest concentration of a chemotactic gradient (1). The permanent presence of microbial and biofilm-derived chemotactic and pro-inflammatory factors attract neutrophils from the circulation into the tissues, where they become activated. Once in the gingival crevice, neutrophils are washed into the oral cavity. Even under healthy conditions, neutrophils constantly infiltrate the periodontium, although in lower numbers than during inflammation. Activated neutrophils have the aim to eliminate or reduce the microbial/antigenic load. The mechanisms by which they contribute to homeostasis and defense are (a) phagocytosis, the ingestion, and intracellular decomposition of antigens, (b) degranulation, the intra- or extracellular release of granule contents such as enzymes and antimicrobial peptides, (c) intra- or extracellular release of reactive oxygen species (ROS), which are directly cytotoxic to microbes but also to host cells, and (d) the formation of neutrophil extracellular traps (NETs), by which neutrophils release decondensed chromatin decorated with histones and granule contents into the extracellular space where they trap microorganisms. All of these processes are closely related, as effective internal degradation of microbes requires intracellular ROS release and degranulation, and as NET formation is usually preceded by ROS formation and accompanied by degranulation (2–5).

Neutrophils are well-researched cells of the innate immune system and yet, new discoveries have been constantly made. Neutrophils are widely believed to be the key cell that mediates tissue destruction and have been described as double-edged swords of immunity. In the periodontium, whilst absence of proper neutrophil function, as seen in some hereditary diseases, like Papillon-Lefèvre syndrome, leads to deleterious periodontal inflammation with severe tissue damage and tooth loss (6), neutrophil hyperactivity is equally associated with tissue damage (7). It has been long noted that neutrophils are heterogeneous in their behavior and perform vastly different functions. For example, only a proportion of neutrophils forms NETs, where the size of that proportion depends upon the stimulus and its concentration (8, 9). Therefore, the question has been raised whether distinct neutrophil subsets may exist and more attention has been given to the research of this possibility.

Potential pro- and anti-inflammatory roles of neutrophil subsets have been demonstrated in recent models of ischaemia-related injury, trauma, cancer, sepsis, and inflammatory diseases (10, 11). Within the oral cavity, different neutrophil subsets and pro-inflammatory neutrophil phenotypes in periodontitis have also been proposed. This mini review aims at summarizing and discussing the current evidence regarding the presence of potential neutrophil subsets and phenotypes in the context of oral health and disease. Those studies are highlighted which have carried out biomarker analysis along with functional assays (Table 1).

**Table 1:** Overview of papers, which have described neutrophil subsets both by their marker expressions and their specific cell functions in the context of oral health and inflammation.

**References**	**Neutrophil test populations, oral health status, and control samples**	**Neutrophil markers described in the test population**	**Functional properties of population**
Wilton et al. (12)	Gingival crevicular fluid neutrophils from healthy donors vs. blood neutrophils	Defective or low CD35	Impaired phagocytosis
Van Dyke et al. (13)	Blood neutrophils from patients with aggressive periodontitis vs. periodontally healthy donors	CD11b^low^	Impaired chemotaxis
Nemoto et al. (14)	Blood neutrophils from patients with aggressive periodontitis vs. periodontally healthy donors	CD16^low^, CD11a^high^, CD11b^high^	Impaired chemotaxis
Miyazaki et al. (15)	Blood neutrophils from patients with chronic periodontitis vs. blood neutrophils	CD16^low^, CD32^low^	Impaired phagocytosis
Kobayashi et al. (16)	Blood neutrophils from patients with chronic periodontitis vs. periodontally healthy donors	CD16b allotype NA2^low^	Impaired phagocytosis and ROS release in response to IgG-opsonized bacteria
Kobayashi et al. (17)	Gingival crevicular fluid neutrophils from patients with chronic periodontitis vs. blood neutrophils	CD89^high^, CD64^high^, CD16b^low^, CD32a^low^	Impaired phago-cytosis of IgG-opsonized bacteria
Fine et al. (18)	Oral neutrophils from patients with chronic periodontitis vs. Para1 and Para2 neutrophils	CD10^high^, CD63^high^, CD64^high^, CD66a^high^ CD11b^high^, CD18^high^, CD55^high^	Increased phagocytosis, NET formation, ROS release, and degranulation
Fine et al. (18)	Oral neutrophils (Para2) from healthy donors vs. Para1 neutrophils	CD55^high^, CD63^high^, CD170^low^, CD16^low^, FSC-A^low^, SSC-A^low^	Increased phagocytosis, NET formation, and unstimulated ROS release
Rijkschroeff et al. (19)	Oral neutrophils from healthy donors vs. blood neutrophils	CD16^high^, CD11b^high^, CD63^high^, CD66b^high^	Increased unstimulated ROS release
Rijkschroeff et al. (20)	Oral neutrophils from edentulous donors vs. dentate donors with no or mild forms of periodontitis	CD16^high^ CD11b^low^, CD63^low^, CD66b^low^	Decreased unstimulated ROS release
Moonen et al. (21)	Oral neutrophils from healthy donors vs. blood neutrophils	fMLPR^low^	Impaired chemotaxis, increased phagocytosis, and unstimulated NET release

*Periodontitis in these studies was diagnosed according to the 1999 classification system (22)*.

## Identification of Neutrophils and Neutrophil Subsets

Traditionally, neutrophils can be readily identified by their typical nuclear shape, in combination with histological hematoxylin and eosin staining (23). Furthermore, on human neutrophils isolated from different sites and in different states of health and activation, 141 cluster of differentiation (CD) markers have been described (24). Mostly, CD11b (integrin subunit alpha M adhesion molecule), CD14 (pattern recognition co-receptor), CD15 (a molecule expressed in the granulocytic series past the myeloblast stage), CD16 (Fc gamma receptor (FcγR) IIIb, low-affinity immunoglobulin (Ig) G receptor), and CD62L (L-selectin, a cell adhesion molecule) (25, 26) have been used individually or in combination to identify pure human neutrophil populations (24). Moreover, several receptors on the neutrophil surface initiate the phagocytic signaling pathway. Phagocytosis is significantly enhanced by the presence of opsonins such as antibodies or complement factors on the microbial surface. Receptors specific for the Fc-region of antibodies include CD64 (FcγRI), CD32 (FcγRIIa), CD16, and CD89 (FcαR, IgA receptor) (27). Furthermore, neutrophils recognize complement-opsonized microorganisms using the complement receptors (CRs) CD35 (CR1), CD11b/CD18 (CR3), and CD11c/CD18 (CR4) (27).

Leliefeld et al. showed that during acute inflammation, a subset of CD16^bright^/CD62L^dim^ hyper-segmented human neutrophils displayed normal phagocytosis, associated with a remarkably poor capacity to contain bacteria intracellularly (28). Conversely, CD16^dim^-banded neutrophils were the only neutrophil subset that adequately contained bacteria. The authors concluded that these results were indicative of a clear neutrophil heterogeneity in their antimicrobial capacity. Several markers of neutrophil degranulation have also been reported. The surface markers are CD63 (primary/azurophilic granules), CD15 and CD66b (secondary/specific granules) as well as CD11b (tertiary/gelatinase granules) and CD13, CD14, CD18, CD45 (quaternary/secretory granules) (29). Another glycoprotein detected within specific granules is Olfactomedin-4 (OLFM4). OLFM4 mRNA has been detected in progenitor neutrophils. However, the protein is only detected in 20–25% of peripheral blood neutrophils (30), suggesting that its expression is regulated by translation mechanisms that lead to functional heterogeneity (31).

The markers described above are not exclusive to neutrophils and have been reported on other cells also, such as macrophages, B- and T-lymphocytes (32). A marker specific to neutrophils is CD177, a glycoprotein expressed on the neutrophil plasma membrane as well as on the membrane of their specific granules (33). However, the proportion of CD177^+^ neutrophils varies amongst individuals, some displaying bimodal (high and no CD177) or trimodal (high, intermediate, and no CD177) expression. Approximately 1–10% of humans show a complete absence of CD177^+^ cells. This has been attributed to the allelic frequency of a mutation in the CD177 gene (31). Due to its heterogeneity, this marker may not be reliable for quantification purposes, but it delineates PMN populations reliably in those individuals who express it. Neutrophil granules have also been used to describe distinct phenotypes: during acute and chronic inflammation a population of neutrophils, which co-sediments with peripheral blood mononuclear cells (PBMCs), has been reported. Typically, circulating neutrophils are separated from PBMCs by gradient centrifugation, as neutrophils have a higher density than PBMCs. Therefore, this neutrophil population has been termed low-density neutrophils (LDNs) (34).

Neutrophils are classically implicated in inflammation, but have some pro-resolving properties also. Firstly, their role as apoptotic cells in the macrophage-mediated resolution of inflammation is believed to be crucial (35). In this process, apoptotic neutrophils induce a suppression of nuclear factor kappa B signaling in macrophages, and therefore a reduction in the release of pro- but not anti-inflammatory cytokines from these cells (36). Secondly, neutrophils are co-producers of the pro-resolving lipid mediators lipoxins and maresin-1 (37, 38).

Thirdly, an immunosuppressive and pro-tumorigenic neutrophil type (N2) has been described previously (39). It features a reduction in Fas-ligand expression, down-regulation of the pathways associated with antigen processing, and a dependency on tissue growth factor beta (TGF-β) signaling (40, 41). Earlier, a similar neutrophil subset with T cell-suppressive properties had been described by Pillay et al. which was mainly based on CD16 and CD62L expression patterns along with nuclear morphology and suppression of T cell proliferation after stimulation with lipopolysaccharide (42). Interestingly, these populations also displayed differences in their survival time and ROS release. Lastly, neutrophils contribute to tissue repair and remodeling, as they produce not only matrix metalloproteinases (MMPs) removing tissue debris at the site of injury, but also tissue inhibitors of MMPs (TIMPs), preventing tissue damage by MMPs, as well as growth factors and pro-angiogenic factors (43). Indeed, a pro-angiogenic neutrophil subset has been observed in human healing wounds. It is characterized by the markers CXCR4, vascular endothelial growth factor receptor 1 (VEGFR1), and integrin alpha subunit CD49d. This subset represented ~3% of total circulating neutrophils (44). These neutrophils produced significantly higher amounts of MMP-9 than pro-inflammatory neutrophils, which was thought to contribute extracellular matrix remodeling at these healing sites, rather than to the extracellular matrix damage mediated by activated inflammatory neutrophils (45).

## Possible Neutrophil Subsets in Periodontal Health and Inflammation

Neutrophils have been identified by means of surface marker detection in high numbers in periodontal tissues (46), gingival crevicular fluid (GCF) (47) and in the oral cavity (24, 27). The presence of oral neutrophils in edentulous subjects has also been demonstrated, suggesting that neutrophils can transmigrate the oral mucosa directly (20). Neutrophils have also been identified within supragingival dental biofilms by means of their CD177 marker (48), indicating that oral PMNs are incorporated into oral biofilms. The functions of those neutrophil surface markers described in the context of periodontal diseases and lined out in Table 1 are explained in Table 2.

**Table 2:** Overview of the physiological functions of the neutrophil surface proteins and markers described in Table 1.

**Neutrophil surface marker**	**Name of surface marker**	**Function of surface marker**
CD10	Membrane metallo-endopeptidase	Cleaves peptides and inactivates several peptide hormones. Involved in neutrophil degranulation.
CD11a, CD11b	Integrin subunits alpha L and alpha M	Mediates leukocyte intercellular adhesion and trans-endothelial migration. Mediates adherence of neutrophils to stimulated endothelium, phagocytosis of complement-coated particles, and degranulation.
CD16	Fc fragment of IgG low affinity receptors IIIa and IIIb	Phagocytosis and clearing of antigen-antibody complexes. Only FcγRIIIb is expressed on neutrophils.
CD18	Integrin subunit beta 2	Combines with multiple different alpha chains to enable cell adhesion and cell-surface mediated signaling during immune responses and cell migration.
CD32, CD32a	Fc fragment of IgG low-affinity receptors II (general) and IIa	Phagocytosis and clearing of antigen-antibody complexes. FcγRIIa is found on phagocytic cells and co-regulates degranulation, FcγRIIb is expressed on B cells and FcγRIIc on a variety of immune cells.
CD35	Complement C3b/C4b receptor 1	Receptors of complement activation. Cellular binding to particles and immune complexes that have activated complement. Involved in neutrophil degranulation.
CD55	Decay accelerating factor for complement	Regulates the complement cascade. Binding to complement proteins accelerates their decay, thereby disrupting the cascade and reducing damage to host cells. Involved in neutrophil degranulation.
CD63	Tetraspanin-30	Mediates signal transduction in the regulation of cell development, growth and motility, and activation including degranulation. Forms complexes with integrins.
CD64	Fc fragment of IgG high-affinity receptor Ia	Phagocytosis and clearing of antigen-antibody complexes.
CD66a, CD66b	Carcinoembryonic antigen related cell adhesion molecule 1 and 8	Cell-cell adhesion, differentiation and arrangement of tissue structure, angiogenesis, apoptosis, tumor suppression, and modulation of immune responses including neutrophil degranulation.
CD89	Fc fragment of IgA receptor	Interacts with IgA-opsonized targets and triggers phagocytosis, antibody-dependent cell-mediated cytotoxicity, release of inflammatory mediators, and neutrophil degranulation.
CD170	Sialic acid binding Ig-like lectin 5	Inhibits the activation of several cell types including neutrophils, involved in cell adhesion, and neutrophil degranulation.
fMLPR (FPR)	Formyl peptide receptor	Receptor for formyl peptides including fMLP. Enhances neutrophil migration and degranulation.

*Source: NCBI (25)*.

Since the 1970s, neutrophil markers in relation to cell function have been investigated in the context of oral health and disease. In 1977, Wilton et al. discovered that only half of the GCF-derived neutrophils isolated from healthy donors expressed the complement receptor C3bR (CD35) and that this led to impaired phagocytosis compared to blood neutrophils (12). However, this assay was based on the assumption that zymosan particles had adsorbed complement factor C3b from serum rather than on identification of CD35. They also reported that the “Fc receptor system” was unimpeded. Later on, in 2000, Kobayashi et al. reported impaired phagocytosis and ROS release in response to IgG-opsonized *Porphyromonas gingivalis* and that these peripheral blood neutrophils from patients with periodontitis displayed a specific FcγRIIIb allotype (NA2) (16). They further reported that GCF-derived neutrophils from these patients had increased FcαRI and FcγRI levels and lower FcγRIIa and FcγRIIIb levels than blood neutrophils, leading to the same impairment of phagocytosis (15, 17).

A neutrophil subset with defective chemotaxis in aggressive periodontitis (Grade 3 periodontitis, according to the 2017 classification scheme (49) was described by Van Dyke et al. (13, 50). The marker, which was diminished on blood-derived neutrophils in these studies, was CD11b (formerly glycoprotein 110), a molecule associated with neutrophil adhesion. Interestingly, the same group reported a diminished capacity of the labeled chemotactic factor N-formylmethionyl-leucyl-phenylalanine (fMLP) to bind to neutrophils in individuals with periodontitis, although the receptors themselves appeared to be functional (51, 52). In line with this finding, a 1997 study by Nemoto et al. demonstrated that blood-derived neutrophils from a similar patient cohort had low CD16, but higher CD11b and CD11b expression levels, and also showed impaired chemotactic capabilities (14). An inability of blood neutrophils from periodontitis patients to migrate in a directional manner and an unresponsiveness to fMLP has also been described elsewhere (53). Furthermore, a hyperactive and hyper-reactive neutrophil phenotype has been reported previously by different groups (7, 54, 55), particularly with regard to ROS release. Although this phenotype has been attributed to higher levels of circulating bacterial products and pro-inflammatory cytokines originating from the periodontium, there is a possibility that these neutrophils may belong to a distinct subset as indicated by later studies, which are described below.

Technological developments over the past decades, particularly with regard to immunofluorescence and flow cytometry methods, have facilitated research of neutrophil markers and subsets, and have led to new insights in recent years. Much attention has been given to oral neutrophils isolated by means of mouth rinsing protocols. A large body of this work has been accomplished by Glogauer and co-workers, who compared neutrophil surface markers in oral and blood neutrophils, as well as in different health and disease states. In 2012, this group identified an oral neutrophil subset in healthy patients that expresses CD3, usually known as a T-cell receptor (56). Although specific interactions between neutrophils and T cells were not investigated in this paper, the finding corroborates the notion that neutrophils act as a bridge between the innate and adaptive immune systems. In 2016, they identified CD11b, CD16, and CD66b as markers that are consistently expressed on neutrophils independent of the cell location, level of activation and periodontal disease state (24), using high-throughput flow cytometry against a panel of 374 known CD antibodies. Furthermore, they newly identified neutrophil surface markers, which had not previously been described. These were CDw198 (C-C chemokine receptor 8), CDw199 (C-C chemokine receptor 9), CD322 (junctional adhesion molecule B, usually known to be localized at tight junctions of endothelial and epithelial cells and to be involved in adhesion and leukocyte transendothelial migration [(25) as well as CD328 sialic acid-binding Ig-like lectin 7, an adhesion molecule (25)].

In the same year, this group reported a CD marker signature indicative of cell activation, which was expressed at much higher levels on oral neutrophils in periodontitis than on healthy oral neutrophils, therefore allowing the distinction between para- and pro-inflammatory neutrophil subsets (18). The para-inflammatory phenotype was defined as neutrophils in an intermediary state that allows them to interact with the oral microflora without eliciting a marked inflammatory response, thus contributing to homeostasis. The markers that were up-regulated on oral neutrophils of periodontitis patients were placed into three categories: markers of activation and degranulation (CD10, CD63, CD64, and CD66a), adhesion receptors (CD11b and CD18) and complement inhibitors (CD55).

The pro-inflammatory phenotype of periodontitis oral neutrophils was confirmed by elevated degranulation, phagocytosis, ROS production, and NET formation. In oral health, the authors observed two different populations of oral neutrophils. These differed in their size and granularity profile, in their expression of specific CD markers, production of ROS, and NET formation. The oral neutrophil phenotype present only in health had a forward scatter and side scatter profile similar to naïve blood neutrophils, and these cells were in a lower state of activation. At the same time, the other, more activated population showed higher expression of CD55 and CD63, whilst having decreased levels of inhibitory receptor CD170 and CD16. The authors concluded that a drop in CD16 in this population is likely a consequence of phagocytosis, and therefore is consistent with elevated phagocytic activity of this activated cell population.

Recently, in 2018, the same group reported that the surface expression of CD63, CD11b, CD16, and CD14 indicates an activation state in oral neutrophils (57), as the presence of these markers implies degranulation, adhesion and antigen recognition processes. They found that oral neutrophil activation was reduced in experimental gingivitis despite higher cell numbers, compared to those seen in health and during the resolution phase. Circulatory neutrophils, on the other hand, were shown to be activated during gingivitis onset as shown by the markers CD55, CD63, CD11b, and CD66a. Also in 2018, this group further characterized the morphology of the para-inflammatory health-associated phenotype described in 2016, and compared it to the naïve morphology of blood neutrophils as well as to the pro-inflammatory phenotype of oral neutrophils in periodontitis (58). They revealed that pro-inflammatory neutrophils in periodontitis showed less granulation, lighter cytoplasms and higher amounts of nuclear euchromatin, as assessed by electron microscopy, compared to the para-inflammatory oral health neutrophils. The periodontitis oral neutrophils also contained more phagosomes with and without undigested bacteria. Neutrophils in gingival tissues displayed naïve morphology when viewed in the blood vessels and gradually showed pro-inflammatory morphological changes as they traveled toward the epithelium.

Another group of researchers led by Nicu and Loos, have also been investigating oral neutrophils by their surface markers (19). They described that in oral neutrophils from healthy donors without additional stimulation, these cells were more activated than circulatory neutrophils, as indicated by higher expression of CD11b, CD63, and CD66b, and elevated constitutive ROS release. The authors concluded that oral neutrophils are in a more mature stage of their life cycle compared with peripheral blood neutrophils, but that they are still responsive to stimulation. Two years later, they published data regarding oral neutrophils from periodontitis patients, and reported that CD11b was elevated in these neutrophils compared to those from healthy controls (59), confirming results from the Glogauer group. Interestingly, this group also investigated oral neutrophils in edentulous patients and found that they expressed low levels of all three activation markers and low constitutive ROS release (20). In a recent publication, these researchers characterized oral neutrophils as terminally migrated cells and reported that oral neutrophils had lost their ability to migrate in a coordinated directional manner (21). They showed that the fMLP receptor, crucial for fMLP-mediated chemotaxis, was detectable in only half of the neutrophils, compared to blood neutrophils. They reasoned that the fMLP receptor was saturated with fMLP from the oral environment and that therefore neutrophils were desensitized to fMLP. In future studies, it would be interesting to investigate whether these receptors are indeed saturated or expressed to a lesser level, as this would give further hints toward a possible novel phenotype of oral neutrophils.

## Conclusion

There is a multitude of reports confirming that blood, tissue, and oral neutrophils act in heterogeneous manners, and that different neutrophil populations and phenotypes exist. It is currently not clear, however, whether neutrophils of the periodontium and oral cavity express or lack certain surface markers in response to stimuli and activation, or whether true subsets exist that shape the specific response of these cells. For example, during the process of transmigration, neutrophils substantially alter their surface marker expression pattern toward higher expression of integrins, chemokines and proteases, and also show delayed apoptosis (10). In this context, the changes in these measurable markers is likely due to their momentary activity rather than reflecting a specific cell subset. At the same time, certain markers such as CD177 appear to be more influenced by the genetic background than by stimuli and cell function. It is also important to consider that oral neutrophils are terminally migrated cells, are under high osmotic stress conditions in saliva compared to blood (60) and are exposed to a high load of microbes, antigens and toxins. These conditions are likely to influence measurable neutrophil parameters, such as granularity, size, release of substances, and possibly the expression of surface markers.

On the other hand, in several systemic diseases, more evidence has been gathered with regard to the existence of true subsets. Studies in the context of oral health and inflammation need to be undertaken bringing together observations of distinct neutrophil populations, markers and behaviors. For example, future studies may be aimed at co-localizing specific CD markers and indicators of neutrophil activity, such as hyper-citrullinated histone for NET formation, or at investigating the influence of different stimuli that activate neutrophils upon the CD marker profile. In many previous studies, flow cytometry has been employed as the main method of neutrophil marker detection, which is a highly suitable tool for this type of research. However, few studies have reported these data in accordance with the MIFlowCyt guidelines proposed by Lee et al. (61). A lack of information regarding gating strategies, instrument settings, and color compensation methods, makes it difficult to critically appraise these data and to reproduce experiments. Therefore, in future studies, these guidelines for reporting flow cytometry data should be adhered to by researchers, which can also help to make data from different research groups more comparable. This seminal field of research has the potential to identify neutrophil populations in the future, which could be utilized as targets in translational research and raise new therapeutic possibilities.

## Author Contributions

JH conceptualized and wrote the article, and prepared the tables.

### Conflict of Interest

The author declares that the research was conducted in the absence of any commercial or financial relationships that could be construed as a potential conflict of interest.
